# Biological Effects Induced by Specific Advanced Glycation End Products in the Reconstructed Skin Model of Aging

**DOI:** 10.1089/biores.2014.0053

**Published:** 2015-01-01

**Authors:** Hervé Pageon, Hélène Zucchi, Zhenyu Dai, David R. Sell, Christopher M. Strauch, Vincent M. Monnier, Daniel Asselineau

**Affiliations:** ^1^ L'Oréal, Research & Innovation, Aulnay-sous-bois, France.; ^2^ Department of Pathology, Case Western Reserve University, Cleveland, Ohio.; ^3^ Department of Biochemistry, Case Western Reserve University, Cleveland, Ohio.

**Keywords:** aging, extracellular matrix, glucose, glycation, methylglyoxal, oxoaldehydes

## Abstract

Advanced glycation end products (AGEs) accumulate in the aging skin. To understand the biological effects of individual AGEs, skin reconstructed with collagen selectively enriched with N^ɛ^-(carboxymethyl)-lysine (CML), N^ɛ^-(carboxyethyl)-lysine (CEL), methylglyoxal hydroimidazolone (MG-H1), or pentosidine was studied. Immunohistochemistry revealed increased expression of α6 integrin at the dermal epidermal junction by CEL and CML (*p*<0.01). Laminin 5 was diminished by CEL and MG-H1 (*p*<0.05). Both CML and CEL induced a robust increase (*p*<0.01) in procollagen I. In the culture medium, IL-6, VEGF, and MMP1 secretion were significantly decreased (*p*<0.05) by MG-H1. While both CEL and CML decreased MMP3, only CEL decreased IL-6 and TIMP1, while CML stimulated TIMP1 synthesis significantly (*p*<0.05). mRNA expression studies using qPCR in the epidermis layer showed that CEL increased type 7 collagen (*COL7A1*), β1, and α6 integrin, while CML increased only *COL7A1* (*p*<0.05). MG-H1-modified collagen had no effect. Importantly, in the dermis layer, *MMP3* mRNA expression was increased by both CML and MG-H1. CML also significantly increased the mRNAs of *MMP1*, *TIMP1*, keratinocyte growth factor (*KGF*), *IL-6*, and monocyte chemoattractant protein 1 (*MCP1*) (*p*<0.05). Mixed effects were present in CEL-rich matrix. Minimally glycoxidized pentosidine-rich collagen suppressed most mRNAs of the genes studied (*p*<0.05) and decreased VEGF and increased MCP1 protein expression. Taken together, this model of the aging skin suggests that a combination of AGEs tends to counterbalance and thus minimizes the detrimental biological effects of individual AGEs.

## Introduction

The aging human skin is characterized by decreased elasticity and accumulation of insoluble collagen and impaired wound healing. These changes are worsened in sun-exposed skin in which proinflammatory changes further help remodel the collagen-rich matrix. Two components are expected to participate in the latter process. The first involves a chemical process in which advanced glycation end products (AGEs) are produced from glucose and oxoaldehydes, thereby inflicting damage to the extracellular matrix, which includes protein crosslinking, insolubilization, and loss of elasticity.^1,2^ The second involves interactions between the modified AGE-rich dermal matrix and dermal cells leading to cell activation via AGE receptors (RAGE) and other receptors, eventually resulting in growth factor and cytokine release that profoundly remodel the ECM.^3^ Many of these changes have been observed in two-dimensional models in which cells are grown onto modified matrix.^3–5^

For several years now, our interest has been to evaluate the role of the aging extracellular matrix in three-dimensional models, that is, the reconstructed skin model in which fibroblasts are embedded in a three-dimensional collagen matrix and establish cross-talk with keratinocytes grown on the dermal matrix.^6^ Using such system, we were able to demonstrate that the glycated matrix mimicked a phenotype that shared many similarities with the aging skin.^7–9^ In particular, we showed that when AGE-rich glycated matrix formed by the reaction of D-ribose with bovine collagen was used, an aging-like phenotype developed. It was notably characterized by the increased presence of N^ɛ^-(carboxymethyl) lysine (CML) in the collagen layer, increased collagen IV in the basement membrane zone, and expansion of α6 and β1 integrins from epidermal to suprabasal layers.^8^ These changes were partly prevented by aminoguanidine and blueberry extract.^8,10^

Given the fact that the composition of AGEs is heterogeneous in the old skin, in this study we sought to define the biological responses of skin reconstructed *in vitro* with specific individual collagen modifications, that is, CML, CEL, and methylglyoxal hydroimidazolones (MG-H1), as well as conditions in which collagen has been relatively enriched with pentosidine, an AGE that accumulates in the aging human skin.^11^

## Materials and Methods

### Preparation of collagen modified by AGEs

#### Preparation of soluble reduced collagen

All AGE-modified collagens used in the study were prepared from collagen that was first reduced with sodium cyanoborohydride at acidic pH in order to prevent conversion of allysine into lysino-norleucine crosslinks. This step was necessary for the preparation of AGE-modified collagen and ensured that collagen would remain in soluble form for the subsequent incorporation of fibroblasts suspended in native collagen mixed in a 1:1 ratio with the modified collagen (see below).

All reactions below were carried out by reverse dialysis under the chemical fume hood in 12-mm-diameter dialysis tubes (MW cutoff 8,000 Da) that contained 30 mL Symatese bovine collagen (4 mg/mL in 0.1 N acetic acid) as supplied by the manufacturer. The tubes were first dialyzed at 4°C against 2 L of 0.5 N acetic acid and then reduced by reverse dialysis for 6 h with the direct addition of 25 g of NaCNBH_3_ (final 100 mM) into the bath while stirring. Tubes were then transferred into a 4 L beaker containing 0.017 N acetic acid and dialyzed twice for 24 h. This preparation was named “reduced collagen” and used as control in biological experiments, and start material for making AGE-modified collagen as described below.

#### Preparation of CML- and CEL-modified type I collagen

Tubes containing “reduced collagen” were transferred into beakers containing 200 mL of Buffer A (Chelex-treated 0.1 M sodium phosphate, pH 7.4, with 1 mM diethylenetriaminepentaacetic acid [DTPA]), with or without glyoxylic acid (100 mM) or pyruvic acid (100 mM) as described.^12^ Reductive alkylation was initiated with the addition of solid NaCNBH_3_ to a final 100 mM concentration and stirred for 4 h at room temperature. All samples were dialyzed in the cold three times for each 24 h against 4 L of 0.5 N acetic acid in order to achieve maximum solubility, and then against 0.1 N acetic acid.

#### Preparation of methylglyoxal-modified collagen rich in hydroimidazolone (MG-H1)

For the preparation of collagen minimally modified by methylglyoxal under oxygen-poor conditions, tubes containing “reduced collagen” were incubated in deaerated Buffer A with or without (control) freshly distilled 1 mM methylglyoxal (MGO) for 24 h at room temperature under nitrogen, followed by dialysis against 0.5 N and then 0.017 N acetic acid in water as above.

#### Preparation of minimally glycoxidized pentosidine-rich collagen

Glycoxidized pentosidine-rich collagen was prepared by incubating for 14 days “reduced collagen” as prepared above with 25 mM D-ribose with added 10 mM N-acetyl-arginine in order to trap free glyoxal release, followed by exhaustive dialysis. All incubations were carried out in metal-free Chelex-100-treated buffer A to which 1 mM DTPA was added. O_2_ was partially removed by bubbling N_2_ gas in order to minimize CML formation.

### Quantification of collagen-linked AGEs by liquid chromatography/mass spectrometry (LC/MS)

An amount of 1.0 mL of the modified collagen was dialyzed against water and freeze-dried. About 1 mg (by weight) was acid hydrolyzed, dried, and taken into 1.0 mL of water for filtration through Spin-x centrifuge filters (Corning Inc.). An amount of 100 μg collagen based on hydroxyproline content was aliquoted and spiked with an isotopically labeled standard mixture as described.^13^ This was dried *in vacuo* and reconstituted to 100 μL in buffer. An amount of 20 μL of this solution was used for analysis (or the equivalent of a 20 μg injection). Carboxymethyl-lysine (CML), carboxyethyl-lysine (CEL), and methylglyoxal-derived hydroimidazolone (MG-H1) were determined in acid hydrolysates of processed collagen samples by electron spray positive ionization-mass spectrometric multiple reaction monitoring (ESI_MRM) using LC-MS/MS system composed of a 2690 Separation module with a Quattro Ultima triple quadrupole mass spectrometry detector (Water-Micromass) following the procedure published by Ahmed and Thornalley.^14^ Equal amounts of collagen (20 μg), whereby collagen content was determined by a hydroxyproline colorimetric assay as described earlier (2), were injected for analysis. Pentosidine was assayed by HPLC as previously described.^15^ All results are expressed as nmol or pmol analyte per mg of collagen.

### Preparation of reconstructed skin containing AGE-modified collagen

Dermal equivalents (fibroblasts contracted collagen gels) and reconstructed skins were prepared as previously described in detail.^16^ Briefly, AGE-modified preparations described above were used for incorporation into the collagen gel. To obtain homogenous AGE-modified collagen lattices, 1 mL of AGE-rich collagen suspension was mixed with 1 mL of fresh collagen (at 3.5 mg/mL 0.017 N acetic acid). After raising the pH (neutral) by addition of 0.1 N NaOH, fibroblasts (1×10^6^ per mL) were added. After 2 h at 37°C the system jellified and started to contract. After contraction (4 days) of the lattice, adult human keratinocytes were seeded onto the lattice and kept submerged for 7 days allowing the cells to form a monolayer. The insert was then raised at the air–liquid interface and kept 1 week to allow the keratinocytes to stratify and differentiate completely. Six samples of both normal and AGE-modified preparations of reconstructed skins were made and studied in the same experiment.

### Histology and immunohistochemistry

Reconstructed skins samples (*n*=4) were fixed in neutral formalin and processed for histology: paraffin sections (5 μm) were stained with hematoxylin–eosin–saffron (HES) or for immunohistochemistry, embedded in Tissue-Tek (Miles Inc.), frozen in liquid nitrogen, and cut into 5-μm-thick sections (cryostat, CM3050 S; Leica Microsystems). Mouse monoclonal antibodies were against human laminin 5 [laminin γ-2 chain] (MAB19562; Chemicon; 1/100). Rat monoclonal antibodies were against human type I procollagen (MAB1912; Chemicon; 1/100) and human α6 integrin (MAB1378; Chemicon; 1/50). Fluorescein isothiocyanate (FITC)-conjugated rabbit anti-mouse immunoglobulins (F232; Dako; 1/100) or FITC-conjugated swine anti-rabbit immunoglobulins (F0205; Dako; 1/50) were used as secondary antibodies. Nuclei were stained using propidium iodide (Sigma). Stained tissue sections were observed and imaged under a fluorescence microscope (DMR; Leica Microsystems). Quantitative image analysis was performed in triplicate using Histolab software (version 7.6.0) from Microvision instruments company.

### Quantitation of growth factors and cytokines in culture medium

The matrix metalloproteinase type 1 (MMP1; RPN2610; Biotrak kit from Amersham Pharmacia), matrix metalloproteinase type 3 (MMP3; Quantikine DMP300; R&D Systems), tissue inhibitor metalloproteinase type 1 (TIMP1; Quantikine DTM100; R&D Systems), vascular endothelial growth factor (VEGF; Quantikine DVE00; R&D Systems), interleukin 6 (IL-6; Quantikine D6050; R&D Systems), and monocyte chemoattractant protein type 1 (MCP-1; Quantikine DCP00; R&D Systems) content of the tissue culture medium were determined using ELISA assays according to the manufacturer's instructions. Six reconstructed skin culture medium samples per condition were analyzed by ELISA (means±SEM are reported).

### Determination of gene expression in collagen matrix

#### RT-PCR

Reconstructed skins (*n*=2) were rinsed in phosphate-buffered saline Dulbecco's without calcium and magnesium (Gibco BRL). Epidermis and dermal equivalents were separated using forceps and frozen. The steps for molecular biology were performed as described below by BioAlternatives Society (Gencay): RNA extraction, reverse transcription, quantitative reverse transcriptase PCR. Marker expression was analyzed by RT-qPCR using mRNA isolated from the samples. Analysis of gene expression was performed with gene-PCR array method. The genes studied for dermis or epidermis are as follows: collagen 1 alpha 1 (*COL1A1*; NM_000088), collagen 3 alpha 1 (*COL3A1*; NM_000090), collagen 7 alpha 1 (*COL7A1*; NM_000094), laminin gamma 2 subunit (*LAMC2*; NM_005562), integrin beta 1 (*ITGFB1*; NM_X07979), integrin alpha 6 (*ITGA6B*; NM_000210), matrix metalloproteinase 1 (*MMP1*; NM_002421), matrix metalloproteinase 3 (*MMP3*; NM_002422), tissue inhibitor of metalloproteinase 1 (*TIMP1*; NM_003254), monocyte chemotactic protein 1 (*MCP1*; M_002982), keratinocyte growth factor (*FGF7*; NM_002009), vascular endothelial growth factor (*VEGF*; M32977), and interleukin 6 (*IL6*; NM_000600), and three housekeeping genes: glyceraldehyde-3-phosphate-dehydrogenase (*GAPDH*; NM_002046), actin beta (*ACTB*; NM_001101), and ribosomal protein L13A (*RPL13A*; NM_012423).

#### Reverse transcription

Total cellular RNA was isolated from the Tris reagent (Sigma-Aldrich)/chloroform mixture by isopropanol precipitation and treated with DNase I (Kit DNase-free; Ambion). RNA was analyzed using Bioanalyser (Agilent Tech). mRNA was reverse-transcribed using appropriate primer oligo(dT) and Supercript II enzyme (Gibco). cDNA was quantified using Nanovue (GE Healthcare) and adjusted.

#### Quantitative PCR

cDNAs were analyzed by quantitative real-time PCR using the LightCycler system (Roche Diagnostics) in duplicate according to the manufacturer's instructions. For each sample, 2.5 μL of cDNA was mixed with appropriate primers and enzymatic kit (LC480 Master SYBR Green 1; Roche) containing taq DNA polymerase enzyme, SYBR Green I marker, and MgCl_2_.

Housekeeping mRNA (GAPDH, RPL13A, ACTB) were quantified in each sample and used for normalization using Rest software version 1.9.12 (Corbett Life Science).

### Statistical analysis

Means and standard errors of the means were determined from four or six samples (respectively, for immunochemistry and ELISA) and data were analyzed using Mann–Whitney nonparametric test. Two reconstructed skin samples were used for molecular biology, which were analyzed with REST software. Significant results are indicated by *p<0.05.

## Results

### Biochemical composition of AGE-modified collagen substrates for reconstructed skin

The composition of the collagens modified either monospecifically by a single AGE or minimally glycoxidized, and therefore enriched with pentosidine and CML, is shown in Table 1. Specific enrichment with CML and CEL was obtained by reductive alkylation with glyoxylic acid and pyruvic acid. About 66% and 5% modification of total lysine residues was modified, respectively. Modification by 1 mM freshly distilled methylglyoxal incubated for 24 h under anaerobic conditions led primarily to formation of the hydroimidazolone MG-H1 (1.4% of total arginine residues were modified) with very little CEL enrichment. While these modifications are supraphysiological, they are similar to those encountered at glycation hotspots and compare well with those often used in similar studies of the biological effects of AGE collagen.^4^ The important consideration for the present study is that the modified collagens highly differ from each other in AGE content, allowing us to probe the significance of each modification *per se*. Finally, we sought to prepare collagen rich in glycoxidation products, in particular pentosidine by incubating with ribose and added N-acetyl arginine under anaerobic and metal-free conditions. Complete suppression of CML by added arginine and anaerobic conditions to prevent CML formation by trapping of glyoxal^17,18^ was not entirely possible, but still highly efficacious.

**Table 1.** Composition of Collagen Preparations Glycated Either Chemically (CML, CEL) or Using Incubation Conditions That Selectively Enrich a Particular AGE (MG-H1, or Pentosidine/CML via Glycoxidation)

Collagen preparation	CML (nmol/mg collagen)	CEL (pmol/mg collagen)	MG-H1 (pmol/mg collagen)	Pentosidine (pmol/mg collagen)
Control	0.67±0.1	83.4±12.2	479.4±114.5	1.36±0.72
CML-rich collagen	**214.8±39.2**	32.2±2.1	427.1±56.3	1.37±0.40
CEL-rich collagen	1.3±0.2	**14,946±240**	357.6±131.4	0.77±0.40
Glycoxidized collagen	9.3±1.2	73.8±8.7	503.4±150.2	**674.5±219.5**
MG-H1-rich collagen	1.5±0.3	73.7±21.0	**6,996±1,314**	0.24±0.51

Bold values were used to indicate the most important AGEs for each collagen preparation.

CML, N^ɛ^-(carboxymethyl)-lysine; CEL, N^ɛ^-(carboxyethyl)-lysine; MG-H1, methylglyoxal hydroimidazolone.

### Expression of proteins, genes, and soluble factors as a function of AGE modification

**FIG. 1.**
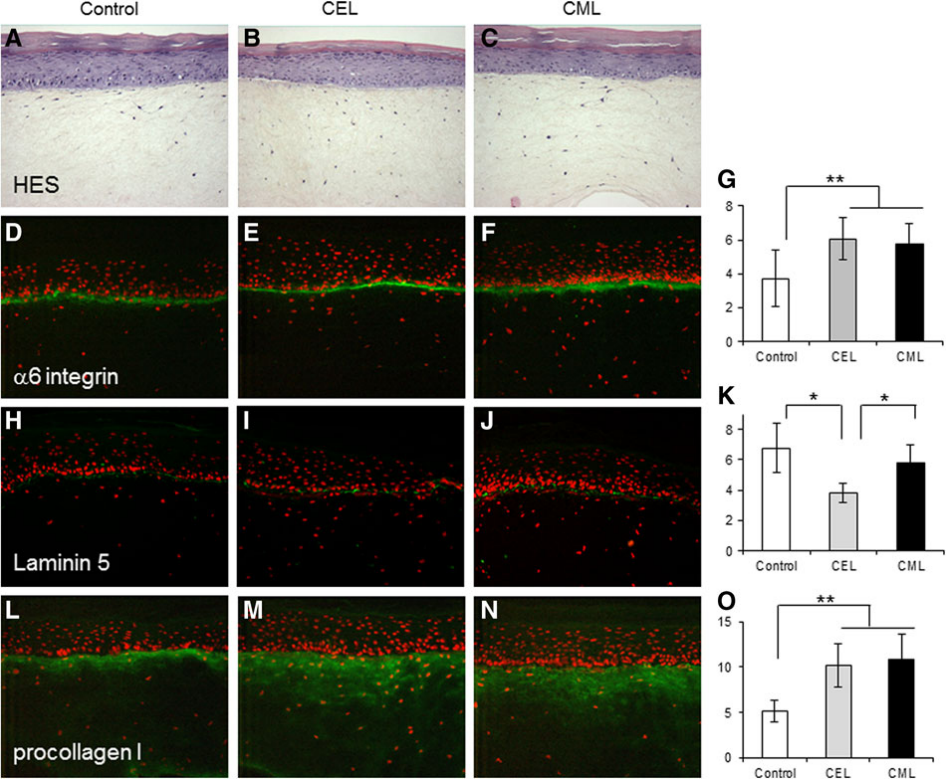
Histology (hematoxylin–eosin–saffron [HES] coloration) and immunostaining of reconstituted skin (*n*=4) prepared with collagen modified by CEL **(B, E, I, M)** and CML **(C, F, J, N)** as compared to control **(A, D, H, L)**. Immunostaining against α6 integrin **(D, E, F)**, laminin 5 **(H, I, J)**, and procollagen I **(L, M, N)**. Surface staining was quantified with picture analysis software: α6 integrin **(G)**, laminin 5 **(K)**, and procollagen I **(O)** for each sample. Values are expressed in arbitrary units (results of three measurements of different zones of each of four samples used for immunochemistry). Means±SD are reported (**p<0.01 and *p<0.05). Integrin α6 and procollagen I are increased by CEL and CML, while laminin 5 is decreased by CEL as compared to control skin.

**FIG. 2.**
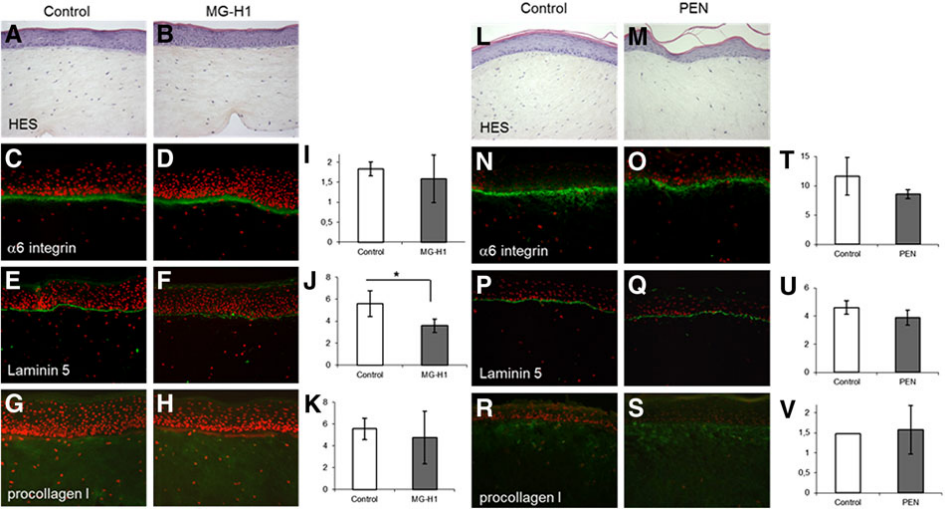
Histology (HES coloration) and immunostaining of reconstituted skin (*n*=4) prepared with methyglyoxal-modified collagen rich in hydroimidazolone: MG-H1 **(B, D, F, H)** as compared to control **(A, C, E, G)**, and glycoxidized collagen (pentosidine [PEN] rich) **(M, O, Q, S)** as compared to control **(L, N, P, R)**. Immunostaining against α6 integrin **(C, D, N,O)**, laminin 5 **(E, F, P, Q)**, and procollagen I **(G, H, R, S)**. Surface staining was evaluated as in Figure 1 for α6 integrin surface **(I, T)**, laminin 5 **(J, U)**, and procollagen I **(K, V)**. Means±SD are reported (*p<0.05). Laminin 5 is significantly decreased by methylglyoxal-modified collagen compared to control.

**FIG. 3.**
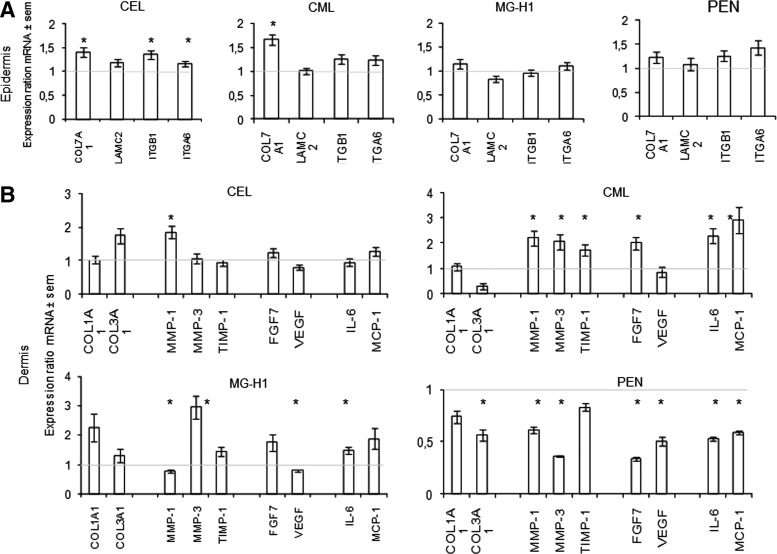
Patterns of gene expression measured by qPCR in epidermal **(A)** and dermal **(B)** layers of reconstructed skin exposed to collagen modified by CML, CEL, MG-H1, and pentosidine (PEN). mRNA levels were quantified in epidermis (keratinocytes) or dermis (fibroblasts) using quantitative reverse transcriptase–polymerase chain reaction at the end of culture emersion phase. Each point represents the mean value of normalized mRNA quantity (*n*=2). qPCR was performed in duplicate on two different samples. Data are expressed in arbitrary units as mean±SEM (*p<0.05).

**FIG. 4.**
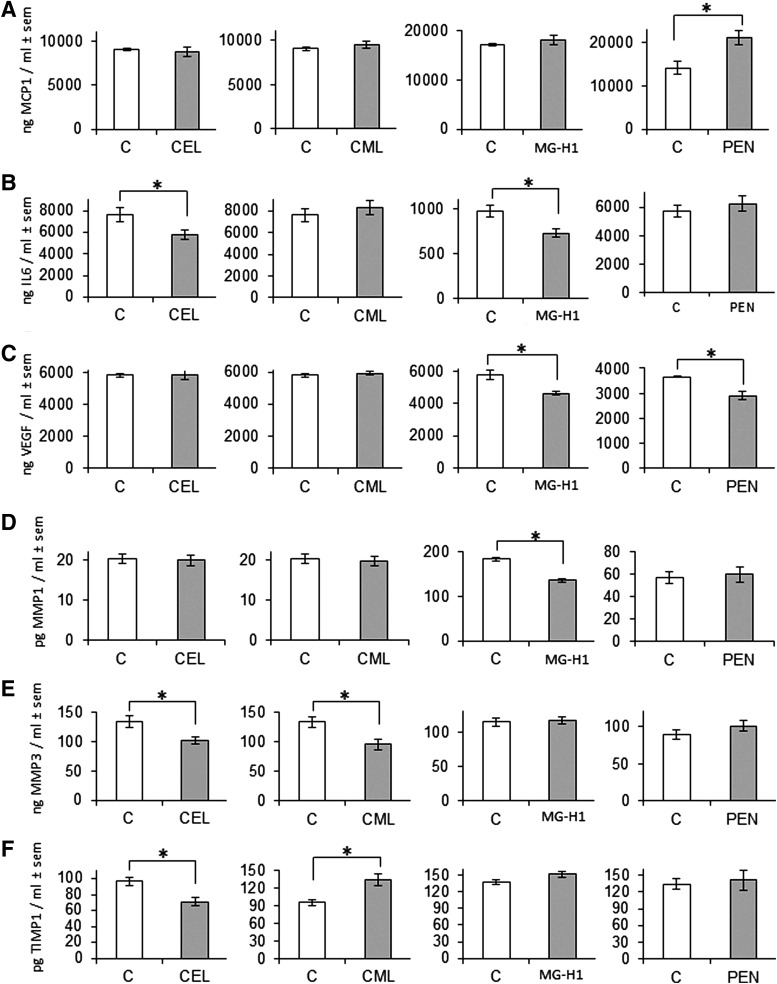
Cytokines, growth factors, and metalloproteinases were assayed by ELISA in the culture supernatant of reconstructed skin (*n*=6) at the end of emersion phase with each collagen preparation (CEL, CML, MG-H1, and PEN). Monocyte chemoattractant protein type 1 (MCP1) **(A)**, interleukin 6 (IL6) **(B)**, vascular endothelial growth factor (VEGF) **(C)**, matrix metalloprotease type 1 (MMP1) **(D)**, matrix metalloprotease type 3 (MMP3) **(E)**, and tissue inhibitor matrix metalloprotease type 1 (TIMP1) **(F)**. Means±SEM are reported (*p<0.05).

The results of the morphological, biochemical, and molecular biological changes observed in the reconstructed skins after exposure to AGE-rich matrix are presented below in Figures 1–4.

### Impact of AGEs on the morphology of reconstructed skin

The histological structure of reconstructed skin observed by HES coloration was not affected by AGE-rich matrix (Fig. 1A–C and Fig. 2A,B,L,M) as compared to the control. Expression of epidermal α6 integrin was increased by CEL and CML (*p*<0.01) compared to control (Fig. 1D–G). Laminin 5 suppression was significantly induced by CEL (*p*<0.05) compared to control or CML (Fig. 1H–K) and also observed with MG-H1 modification (*p*<0.05) (Fig. 2E–J). We observed a robust increase (*p*<0.01) in procollagen I expression by CML- and CEL-rich matrix (Fig. 1L–O) but no effect of MG-H1 and pentosidine (Fig. 2G–K, R–V, respectively). Pentosidine-rich collagen tended to suppress α6 integrin and laminin 5, but the effects were nonsignificant (Fig. 2N–U).

### Patterns of gene expression in response to collagen modification by CML-, CEL-, MG-, H1-, and pentosidine-rich collagen

The reconstructed skins were separated into dermal and epidermal layers, each of which was used for quantitation by qPCR of an array of genes pertinent to skin aging and remodeling.

The data in Figure 3 are expressed as a ratio between AGE-modified collagen and the corresponding control. In the epidermal cells (Fig. 3A), CEL increased type 7 collagen (*Col7A1*), β1 integrin (*ITGB1*), and α6 integrin (*ITGA6*), while CML only increased *COL7A1* significantly. No significant alterations were observed with MG-H1 and pentosidine-rich collagen.

In the dermal cells (Fig. 3B) CML increased significantly *MMPs*, *TIMP1*, *FGF7* (*KGF*), *IL6*, and *MCP1* (*p*<0.05) and seemed to upregulate the majority of genes studied. Genes studied were not affected by CEL except for upregulation for *MMP1* (*p*<0.05). Methylglyoxal hydroimidazolone MG-H1 modulated most genes with robust upregulation of *MMP3* and some increase of *IL6* (*p*<0.05) and slight downregulation of *MMP1* and *VEGF* (*p*<0.05). Interestingly, minimally glycoxidized pentosidine-rich collagen suppressed significantly most mRNAs studied (*p*<0.05). Two opposite effects seem to be observed with CML- and pentosidine-rich collagen concerning the mRNA regulation.

### Release of cytokines, growth factors, and proteases into the culture medium in response to CML-, CEL-, MG-, H1-, and pentosidine-rich collagen

Because changes in mRNA expression are not necessarily accompanied by changes in protein expression, we sought to quantitate relevant cellular factors and matrix remodeling molecules and cytokines that are secreted into cell culture medium. Growth factors (VEGF and KGF) and molecules implicated in the inflammatory process (IL-6 and MCP1) and matrix remodeling (MMP1, MMP3, and TIMP1) were quantified in the culture medium (Fig. 4). No change in KGF release was observed with any collagen modifications (data not shown). However, it is interesting to note that VEGF was repressed by MG-H1 and pentosidine-rich modified collagen (*p*<0.05) while unaffected by CEL or CML (Fig. 4C). CML was the only collagen modification with no effect on the molecules implicated in the inflammatory process. Indeed, both CEL and MG-H1 decreased IL-6 expression (*p*<0.05) (Fig. 4B), while pentosidine-rich collagen stimulated MCP1 expression (*p*<0.05). Regarding the molecules implicated in matrix remodeling (Fig. 4D,E), both CML and CEL significantly suppressed MMP3 release (*p*<0.05) (Fig. 4E). In contrast, tissue inhibitor of metalloproteinase (TIMP1) (Fig. 4F) was stimulated by CML and repressed by CEL (*p*<0.05). MG-H1-modified collagen seems to decrease only the release of MMP1 (*p*<0.05) (Fig. 4D).

## Discussion

Understanding the mechanisms that underlie skin aging is of importance not only for cosmetic purposes and the fight against the stigma of old age, but also from a biomedical viewpoint considering that the old skin is more prone to impaired wound healing, ulceration, infection, autoimmune diseases, and various malignancies.^19^ One of the hallmarks of aging skin, whether sun exposed or not, is the accumulation of AGEs. A recent study revealed that 250 mol% of collagen residues in skin are damaged by advanced glycation at 80 years of age.^20^ Moreover, AGE levels increased two- to fourfold in diabetes, and the biologically active AGEs and proinflammatory RAGE ligand CML are dramatically increased in sun-exposed skin.^21^ Given that it takes 15 years for 50% of skin collagen to turn over,^22^ there has been a pragmatic interest for understanding the relationship between advanced glycation and the biology of the modified extracellular matrix and its role in intrinsic skin aging and age-related skin diseases.

The above study provides the first systematic and comparative insight into the roles of the collagen-linked glycoxidation products CML, CEL and pentosidine, and the methylglyoxal-derived MG-H1 in the expression of extracellular matrix genes, angiogenesis, and nonimmune response-related cytokines in the reconstructed three-dimensional skin model. The study reveals a clustering of similar responses to modifications of lysine free amino groups, that is, CML and CEL, versus those that also modify arginine residues, that is, pentosidine (a lysine–arginine crosslink) and MG-H1. The latter oxoaldehyde predominantly modifies arginine residues. Thus, both CML and CEL modifications of collagen induced α6 integrin and procollagen I protein expression (Fig. 1). The data suggest that fibroblasts either sense the blocked primary amines from collagen or perceive the negative charge as a signal to increase ECM synthesis and α6 integrin expression for better attachment of the cell to the ECM. In contrast, both MG-H1 and pentosidine-rich collagens tended to decrease several ECM markers, though significance was reached only for laminin 5 suppression by methylglyoxal modified collagen (Fig. 2). In addition, pentosidine-rich collagen significantly downregulated most ECM genes studied (Fig. 3B).

Since laminin 5 was also markedly suppressed by CEL (*p*<0.05) and more mildly by CML (no significantly), it would appear that the loss of positive charges on collagen, either from lysine or arginine, is a common signal for downregulation of laminin 5 synthesis. In contrast, while methylglyoxal-modified collagen also significantly suppressed laminin 5 expression, it had no or minimally suppressive effects on α6 integrin and procollagen I expression (Fig. 2C–K). Very similar effects were noted with glycoxidized, pentosidine-rich collagen (Fig. 2N–V). Thus, in contrast to pure lysine modifications, arginine modifications overall tended to suppress rather than enhance the expression of these critical extracellular matrix proteins. This is an exciting finding, as it suggests that the combination of AGEs targeting lysine and arginine residues might have the effect of mutually cancelling out the deleterious biological effects resulting from individual AGEs.

The extent to which the above results are RAGE dependent has not been investigated here. Fibroblasts grown on CML-rich collagen undergo apoptosis^23^ and CML is a ligand for RAGE.^24^ Unlike in the present study, collagen synthesis by fibroblasts was suppressed after exposure to AGE β2 microglobulin by a mechanism that was partly RAGE dependent.^25^ However, the findings of very low levels of RAGE expression by fibroblasts^26^ do not support an important role for RAGE in the above results. Similarly, there was no induction of mRNA RAGE expression in keratinocytes cultured in procollagen I or III and fibronectin.^27^

The disproportionate upregulation of integrin versus downregulation of laminin 5 in CML- or CEL-exposed matrix raises the question whether keratinocytes experience attachment problems. Indeed laminin 5 (laminin 332) is an essential component for linking the epidermal basal cells to the papillary dermis, for the resistance of epidermis to external stress and for building basement membrane at the dermal epidermal junction.^28^ α6β4 is one of the specific receptors for laminin 5.^29^ Thus, the α6 integrin increase observed with CEL and CML could serve to compensate for the laminin 5 decreases. The impairment of basement membrane structure may be associated with functional changes of cells and facilitate aging process by damaging dermal extracellular matrix and inducing keratinocyte abnormality.^30^

The same phenomenon may apply to methylglyoxal-treated matrix. Indeed data from Dobler et al. revealed increased anoikis presumably linked to blocking of ECM protein RGD sequences and β integrin attachment.^31^ Alikhani et al. reported similar apoptosis induction of CML-rich collagen subcutaneously implanted into rat.^4^

Another important paradigm for the aging skin is that CML and CEL modifications are both associated with increased *MMP1* and *MMP3* mRNA expression in the dermis (see also Fig. 3). Indeed considerable amount of data implicate MMP1 upregulation in the aging skin.^32^ One mechanism by which this may happen is by engagement of the RAGE by collagen-bound CML and CEL. RAGE, a proinflammatory receptor, is constitutionally expressed in fibroblasts.^33^ Thus, this could suggest that fibroblasts attempt to degrade the highly modified matrix, while keratinocytes try to attach to the latter. TIMP-1 was generally not increased, except by CML-rich matrix (Fig. 3B and 4F). Tissue inhibitor of metalloproteinase or TIMP-1 is a natural inhibitor of several MMPs.^34^ Thus, CML might attempt to counterbalance matrix degradation during aging skin process through increasing TIMP1. The deregulation of the balance between MMPs and TIMPs is involved in the matrix degradation process in skin aging.^35^ Similarly, the increased production of procollagen I (Fig. 1L–O), *COL3A1* (CEL), and *COL1A1* (MG-H1) (Fig. 3B) can also be interpreted as an attempt to produce fresh unmodified collagen for better cell attachment or for regenerating ECM.

The release of most of the tested inflammatory markers (MCP-1, IL-6) and growth factors (KGF, VEGF) into the medium was not increased by the modified collagens. It was rather suppressed for IL-6 (CEL, MG-H1) and VEGF (MG-H1, pentosidine), except for pentosidine-rich collagen that stimulated MCP-1 secretion and could impart a potential proinflammatory effect. However, most mRNAs coding for matrix component or soluble factor were downregulated in minimally glycoxidized, pentosidine-rich collagen. These genes are involved in many biological processes, for example, *IL-6* in wound healing,^36^ *VEGF* in angiogenesis,^37,38^ and *MCP1* in matrix protein synthesis,^39^ and wound repair during aging.^40^ In addition, reduced VEGF production could cause a decline in angiogenesis bringing about the vessel disappearance observed during skin aging^41^ and eventually the disappearance of dermal papillae.^42^ Thus, any alterations of production and regulation of these factors could play an important role in skin homeostasis.

The signaling processes leading to increased production of procollagen I by CML and CEL remain to be defined. Procollagen expression is controlled by various complex mechanisms.^43^ However, secreted IL-6 does not appear to be involved since it was suppressed by CEL and MGO-modified matrix, though its mRNA was increased in the dermis layers of CML and MGO-modified matrix. Thus, clearly, the biological response to modified matrix is not uniform and in-depth studies will be needed to decipher the factors that control matrix composition and remodeling as a function of alteration induced by specific AGEs.

A word of caution is necessary concerning extrapolation of our data to the *in vivo* aging human skin. Levels of our AGEs prepared by glycation were generally comparable with those in an 80-year-old skin (excepted for pentosidine). Levels expressed in pmol/mg collagen in bovine versus human were about 670 versus 400–600 (CML), 84 versus 300 (CEL), 479 versus 800 (MG-H1), and 1.36 versus 30 (pentosidine). However, to study individual AGEs prepared by alkylation, that is, CML and CEL, supraphysiological levels were used in order to boost potential biological responses. Similarly, while the reconstructed skin model is a major improvement over culture dishes, absence of vessels and inflammatory cells limits its usefulness for the study of pathological processes. However, like the normal aging skin that has very few inflammatory cells, our data suggest that the reconstructed skin is adequate to model biological processes of the non-sun-exposed aging skin.

In summary, we have shown that individual AGEs exert differential effects on the homeostasis of the reconstructed skin. For the first time, we achieved a comparative study between various products of glycation in a tridimensional skin model. It appears that AGEs do not have the same deleterious impact during *in vitro* skin aging. Some might even have beneficial properties to counteract the deleterious properties from others, such as the futile attempt to upregulate MMP1 and MMP3 via increased mRNA expression. However, the data also support strong detrimental effects of selected AGEs on tissue remodeling that favor accumulation of matrix and impaired angiogenesis, all of which are hallmarks of the aging skin.

## Abbreviations Used

CEL: N^ɛ^-(carboxymethyl)-lysine
DTPA: diethylenetriaminepentaacetic acid
KGF: keratinocyte growth factor
MG-H1: methylglyoxal hydroimidazolone
MMP1: matrix metalloproteinase type 1
RAGE: advanced glycation end product receptors
TIMP1: tissue inhibitor metalloproteinase type 1
TIMP1: tissue inhibitor of metalloproteinase
VEGF: vascular endothelial growth factor
